# Supplementary material for: Effect of Combining Wuyiencin and Pyrimethanil on Controlling Grape Gray Mold and Delaying Resistance Development in Botrytis cinerea
Source: Microorganisms. 2024 Jul 8;12(7):1383. doi: 10.3390/microorganisms12071383 (PMC11279109; doi:10.3390/microorganisms12071383)
Supplement: Supplementary file 1 [file microorganisms-12-01383-s001.zip › microorganisms-3080859-supplementary.pdf]

# Effect of Combining Wuyiencin and Pyrimethanil on Controlling Grape Gray Mold and Delaying Resistance Development in *Botrytis cinerea*

**Jiabei Xie [1,†], Boya Li [1,2,†], Jia Li [3], Kecheng Zhang [1], Longxian Ran [2] and Beibei Ge [1,*]**

[1] State Key Laboratory of Biology of Plant Diseases and Insect Pests, Institute of Plant Protection, Chinese Academy of Agricultural Sciences, Beijing 100093, China; xiejiabei1998@163.com (J.X.); l18703242721@163.com (B.L.); kczhang@ippcaas.cn (K.Z.)
[2] College of Forestry Sciences, Hebei Agricultural University, Baoding 071000, China; longxianran@163.com
[3] State Key Laboratory of Biocatalysis and Enzyme Engineering, School of Life Sciences, Hubei University, Wuhan 430062, China; lijia@hubei.edu.cn
[*] Correspondence: gebeibei@caas.cn
[†] These authors contributed equally to this work.

Figure S1 Regression analysis of wuyiencin toxicity on the colony growth of *Botrytis cinerea*

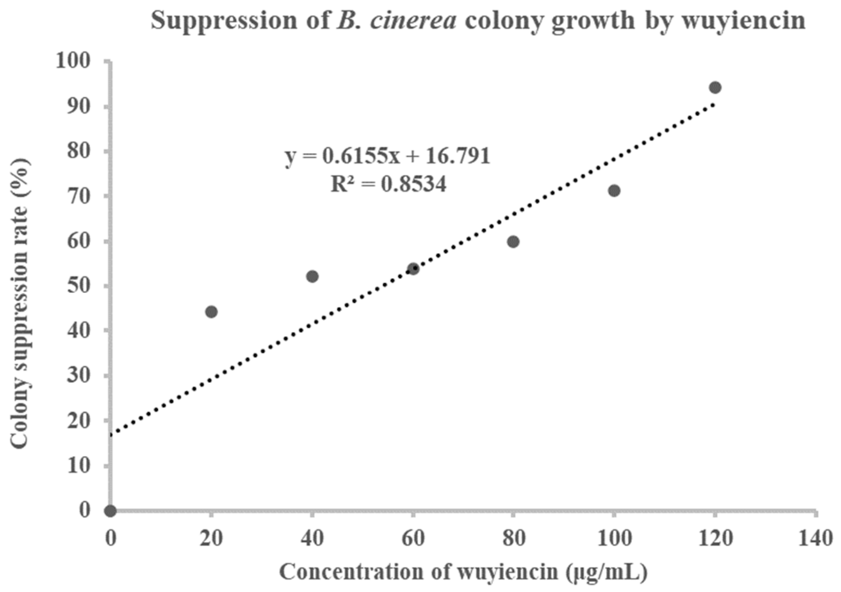

Figure S2 Effects of wuyiencin on hyphal morphology of *Botrytis cinerea*

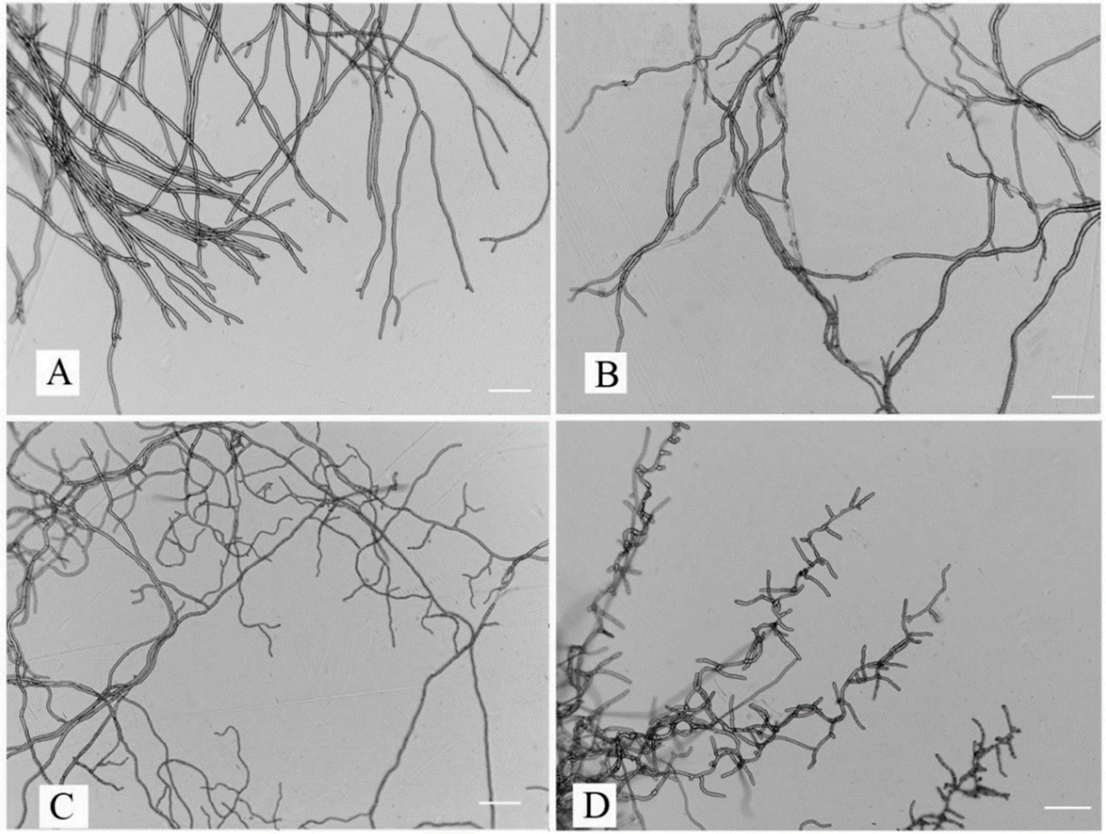


Note: A, B, C, and D show effects of wuyiencin concentrations of 0 (control), 20, 60, and 100 µg mL$^{-1}$, respectively. Images were taken 5 d after treatment; scale bar = 100 µm.

Figure S3 Effects of wuyiencin treatment on conidial germination of *Botrytis cinerea*

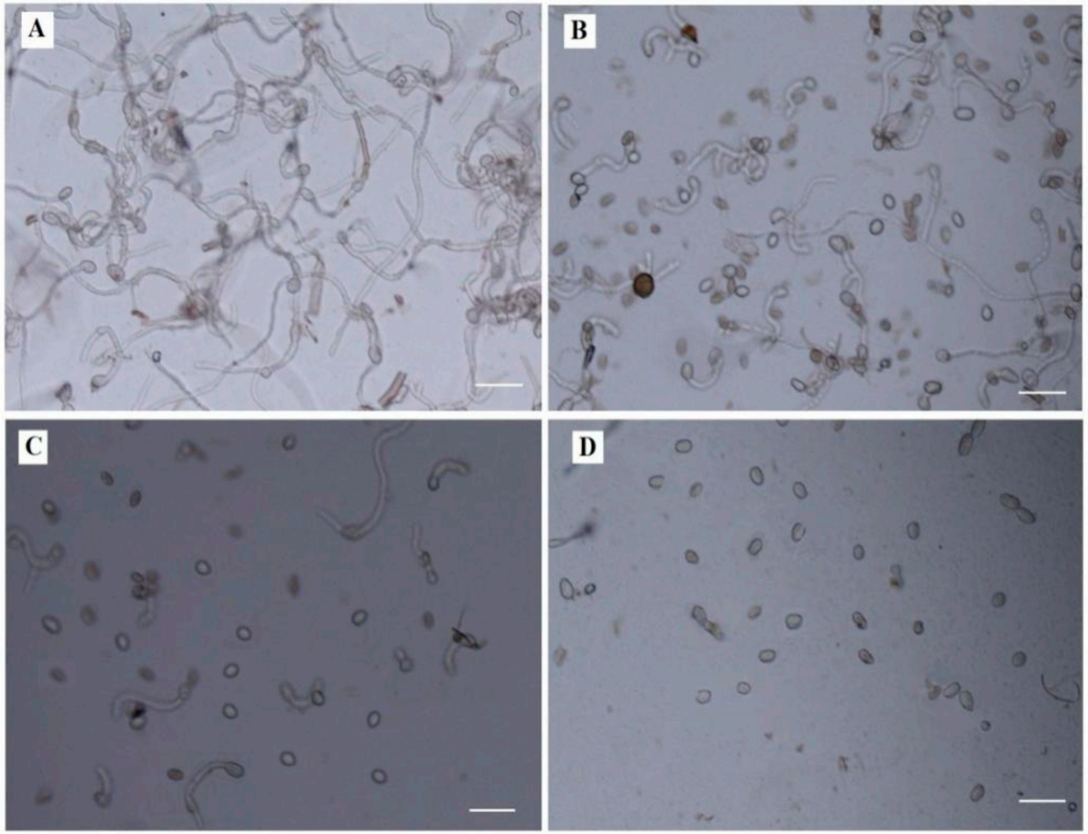


Note: A, B, C, and D show effects of wuyiencin concentrations of 0 (control), 20, 60, and 100 µg $mL^{-1}$, respectively. Images were taken 24 h after treatment; scale bar = 100 µm.

Figure S4 Effects of wuyiencin treatment on infection pad formation of *Botrytis cinerea*

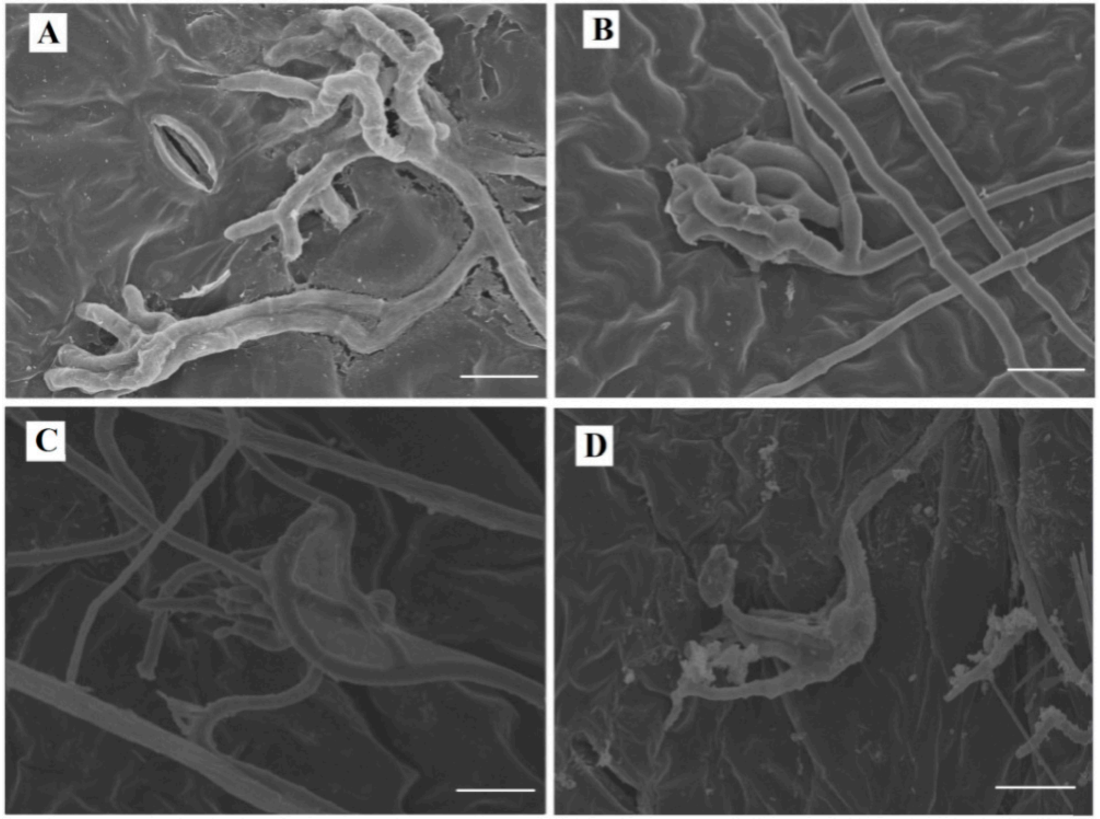


Note: A, B, C, and D show effects of wuyiencin concentrations of 0 (control), 20, 60, and 100 µg $mL^{-1}$, respectively. Leaves were inoculated 24 h after wuyiencin treatment, and the scanning electron microscopy images were taken 4 days after wuyiencin treatment; scale bar = 10 µm.

Figure S5 Effects of wuyiencin treatment on oxalic acid production of *Botrytis cinerea*

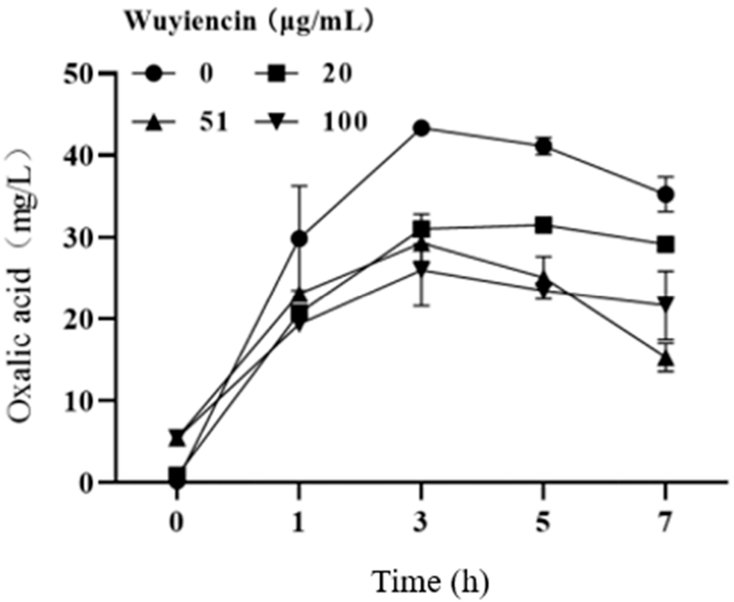

Figure S6 Effects of wuyiencin on callose deposition in grape leaf tissues

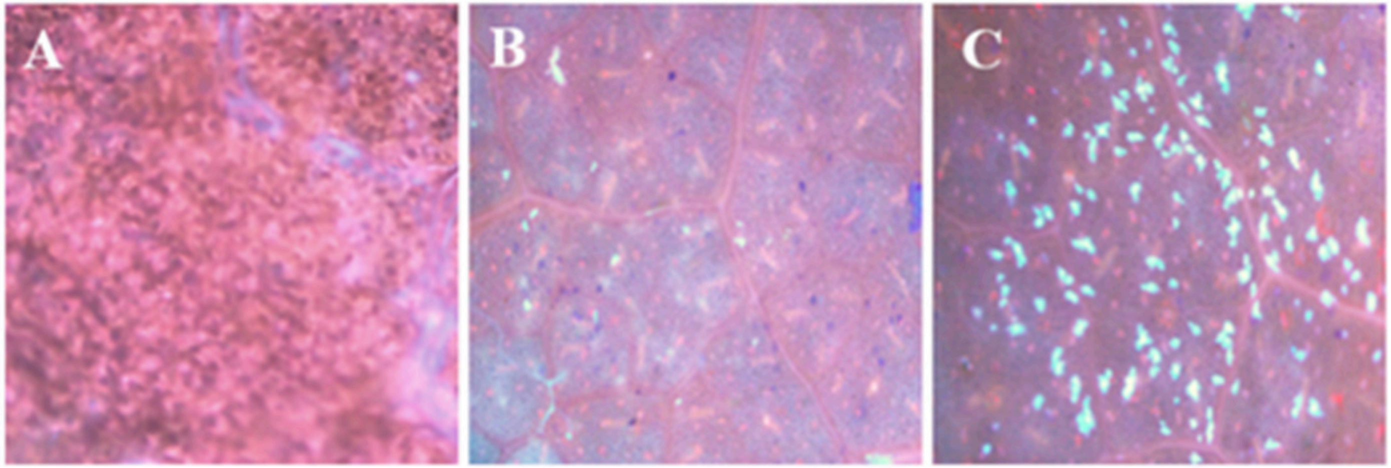


Note: A, no inoculation of *Botrytis cinerea* and no wuyiencin treatment; B, inoculation with *B. cinerea* but no wuyiencin treatment; C, inoculation with *B. cinerea* and wuyiencin treatment.

Images were taken 24 h after inoculation with *B*. *cinerea*, which was performed 24 h after wuyiencin application; scale bar = 100 µm.

Figure S7 Inhibitory effect of different concentrations of various fungicides on *Botrytis cinerea* colony growth

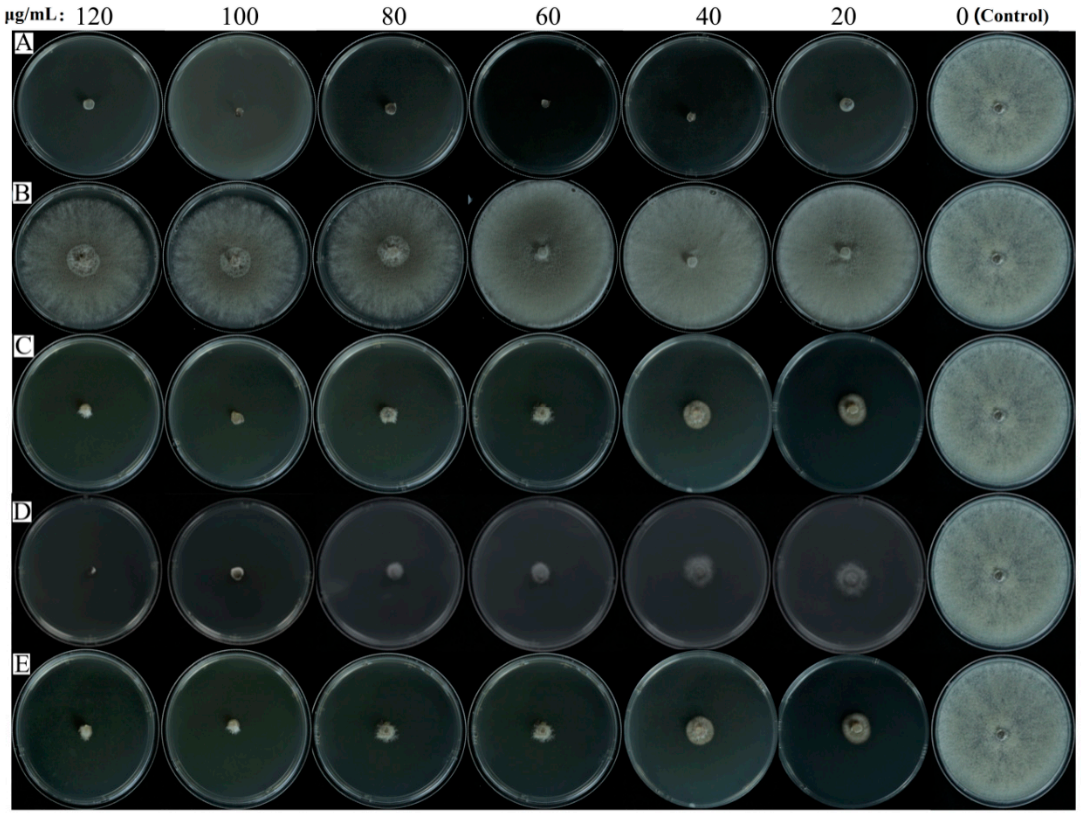


Note: Images were taken 3 days after inoculation with mycelial discs (6 mm in diameter) of *B. cinerea*. The potato dextrose agar plates contained wuyiencin (A), polyoxin (B), iprodione (C), pyrimethanil (D), and fluopyram/trifloxystrobin; (E) Each fungicide was used at the following concentrations: 0 (control), 20, 40, 60, 80, 100, and 120 μg $mL^{-1}$.

Figure S8 Inhibitory effect of the optimal mixed preparation of fungicides on *Botrytis cinerea* colony growth

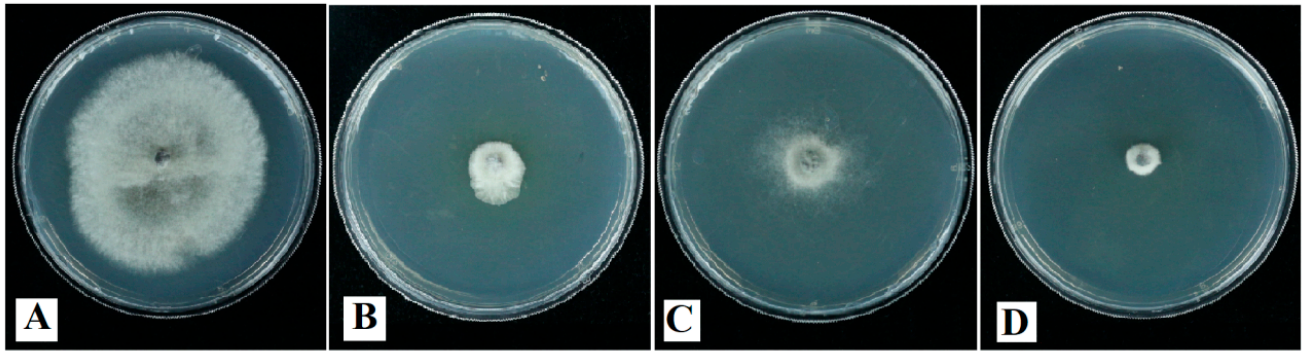


Note: Images were taken 3 days after inoculating mycelial discs (6 mm in diameter) of *B. cinerea* on potato dextrose agar. A, B, C, and D depict the control group and wuyiencin $EC_{50}$, pyrimethanil $EC_{50}$, and the mixed preparation (wuyiencin $EC_{50}$: pyrimethanil $EC_{50}$ = 7:3) treatment groups, respectively.

Table S1 Fungicides used in the present study

| Fungicide name | Effective Cont. (g $L^{-1}$) | Formulation type | Manufacturer |
|---|---|---|---|
| Pyrimethanil | 400 | Suspension | Bayer China Co. Ltd. |
| Iprodion | 500 | Suspension | • Fumei Industrial Co., Ltd. |
| Polyoxin | 100 | Wettable powder | Japan Res. Pharmacy Co. |
| Fuopyram•trifloxystrobin | 430 | Suspension | Bayer China Co. Ltd. |
| Wuyiencin | 80 | Suspension | IPP-CAAS |

Table S2 Pathogenicity-related genes of *B. cinerea* for studying their expressions and primers designed

| Gene name | Pathogenicity-related function | Primer sequences (5′ to 3′) | Gene sequence (bp) | Reference |
|---|---|---|---|---|
| Transmembrane proteingene (*Bcpls*) | Appressorium formation | GGGGATGGTCTAAGGTGTTG<br>TTATGCTGCTCCTGCTCTGG | 228 | [64] |
| MAP kinase gene (*Bmp1*) | Leaf infection | AGCAGCGTCAGGTTGAAATG<br>GGCAATGGTCGTCGGATAAA | 109 | [65] |
| NADPH oxydase gene (*BcnoxA*) | Pathogenicity development | CGAAACTAACAGACTACAGATGCC<br>CCGACTTGACGGATGTGGAT | 179 | [66] |
| Endopolygalacturonase gene (*Bcpg1*) | Pectin degradation | GCGGGGCTAGGAGTGTTTTA<br>GCAGCGAGCCATAGGAGTTT | 139 | [67] |
| Actin gene (*Bcact*) | Cell development<br>(as internal control reference) | AGCGTGAAATCGTCCGTGAT<br>GACTGGCGGTTTGGATTTCTT | 81 | [68] |

Note: The gene sequences were consulted from NCBI GenBank and the primers were designed based on theses gene sequences with the Primer 5 software.

Table S3 Inhibition effect of wuyiencin at different concentration on *B. cinerea* growth

| Wuyiencin concentration (μg mL$^{-1}$) | Colony growth | | Conidia germination | |
|---|---|---|---|---|
| | Diameter (mm) | Inhibition (%) | Germination rate (%) | Inhibition (%) |
| 0 (Control) | 65.0±2.6a | 0 | 97.2±4.2a | 0 |
| 20 | 36.0±6.0b | 44.6 | 61.8±3.8b | 36.4 |
| 40 | 31.0±6.8b | 52.3 | 48.3±3.5c | 50.3 |
| 60 | 29.9±2.4b | 54.0 | 20.4±2.1d | 72.8 |
| 80 | 26.0±2.9b | 60.0 | 8.6±0.8e | 91.2 |
| 100 | 18.7±6.7c | 71.2 | 3.9±0.9f | 96.0 |
| 120 | 3.7±1.1d | 94.3 | 2.1±0.5f | 97.8 |

Note: The data were presented as mean ± SD of 3 replicates; different letters indicate numbers are significantly different at $P<0.05$. The inhibition percentages were computed from the mean diameters.

Table S4 Suppression effect of wuyiencin on gray mold (*B. cinerea*) on grape leaves and fruits

| Wuyiencin (μg $mL^{-1}$) | Gray mold of grape fruits | | Gray mold of grape leaves | |
|---|---|---|---|---|
| | Lesion diameter (cm) | Disease suppression rate (%) | Disease incidence (%) | Suppression rate (%) |
| 0 (control) | 5.9±0.4a | 0.0 | 98.3±2.9 a | 0.0 |
| 50 | 4.8±0.4b | 18.5 | 84.2±3.8 b | 14.3 |
| 80 | 2.5±0.2c | 57.6 | 52.5±2.5 c | 46.6 |
| 100 | 1.9±0.2d | 67.8 | 10.0±4.3 d | 89.8 |
| 120 | 0.7±0.2e | 88.1 | 3.3±2.9 e | 96.6 |

Note: The lesion diameter data are the means of 9 lesions with standard errors and different letters after the data indicate that they are significantly different at $P<0.05$ analyzed with Duncan's new multiple range test method.

Table S5 Suppression effect of the selected best mixed preparation on colony growth of *B. cinerea*.

| Fungicide | Colony diameter (cm) | Suppression rate (%) |
|---|---|---|
| Control | 6.24±0.68 a | 0.00 |
| Wuyiencin $EC_{50}$ | 1.49±0.31 c | 76.12 |
| Pyrimethanil$EC_{50}$ | 3.11±0.63 b | 50.16 |
| Wuyiencin $EC_{50}$+Pyrimethanil $EC_{50}$(7:3) | 1.26±0.16 c | 79.81 |

Note: After 4 days the colony diameter was measured. Each diameter datum is the mean of 3 replicates with standard error and different letters after the data indicate that they are significantly different at $P<0.01$ tested with Duncan's new multiple range test method.
